# Automatic Post-Stroke Severity Assessment Using Novel Unsupervised Consensus Learning for Wearable and Camera-Based Sensor Datasets

**DOI:** 10.3390/s23125513

**Published:** 2023-06-12

**Authors:** Najmeh Razfar, Rasha Kashef, Farah Mohammadi

**Affiliations:** Department of Electrical, Computer, and Biomedical Engineering, Faculty of Engineering and Architectural Science, Toronto Metropolitan University, Toronto, ON M5B 2K3, Canada; nrazfar@torontomu.ca (N.R.); fmohamma@torontomu.ca (F.M.)

**Keywords:** stroke, Wearable Sensor (Xsens), camera-based system, automated assessment, level of severity, clustering, consensus clustering, trunk displacement

## Abstract

Stroke survivors often suffer from movement impairments that significantly affect their daily activities. The advancements in sensor technology and IoT have provided opportunities to automate the assessment and rehabilitation process for stroke survivors. This paper aims to provide a smart post-stroke severity assessment using AI-driven models. With the absence of labelled data and expert assessment, there is a research gap in providing virtual assessment, especially for unlabeled data. Inspired by the advances in consensus learning, in this paper, we propose a consensus clustering algorithm, PSA-NMF, that combines various clusterings into one united clustering, i.e., cluster consensus, to produce more stable and robust results compared to individual clustering. This paper is the first to investigate severity level using unsupervised learning and trunk displacement features in the frequency domain for post-stroke smart assessment. Two different methods of data collection from the U-limb datasets—the camera-based method (Vicon) and wearable sensor-based technology (Xsens)—were used. The trunk displacement method labelled each cluster based on the compensatory movements that stroke survivors employed for their daily activities. The proposed method uses the position and acceleration data in the frequency domain. Experimental results have demonstrated that the proposed clustering method that uses the post-stroke assessment approach increased the evaluation metrics such as accuracy and F-score. These findings can lead to a more effective and automated stroke rehabilitation process that is suitable for clinical settings, thus improving the quality of life for stroke survivors.

## 1. Introduction

Stroke is the third major cause of disability. As reported by disability-adjusted life years lost [DALYs], 143 million cases of disability worldwide in 2019 were due to stroke. Nearly 60% of post-stroke patients with upper-limb hemiparesis in the severe stage experience chronic major functional impairment [1]. Rehabilitation is highly recommended for stroke survivors as one of the most effective treatments to accelerate motor functions in the affected parts of their bodies [2,3]. The first step in a rehabilitation process is the assessment of the affected body part for rehabilitation planning and strategy. Traditionally, such assessments are self-report-based or conducted according to an expert decision. The Fugl-Meyer Assessment (FMA) is one such assessment in which clinicians measure sensorimotor impairment and functional movements of the body associated with the range of motion, muscles, and joints. It also measures levels of severity in stroke survivors. FMA-UE refers to the FMA score for the Upper Extremities, which consists of 33 tasks scored between 0 and 2 points [2]. If a patient performs the required task fully or partially, or is unable to perform the task, the assigned point value will be 2, 1, or 0, respectively. The sum of the points from all tasks will be the FMA score assigned for the patient, which ranges between 0 and 66. The used measures are manually calculated using a checklist approach, based on the clinician’s opinion; however, this is a time-consuming and subjective process [4,5]. Moreover, the visual assessment involves some extent of doubt from various sources, such as from assessment gratitude [6] or motion irregularity [7,8,9]. Defining the patients’ movements accurately with comparable performance indicators will positively impact the target planning of rehabilitation strategies [10,11]. Therefore, automating this assessment will benefit both the rehabilitation process as well as the strategy and planning for regaining movement, which in turn would facilitate the stroke assessment process [9]. Automating assessment scores can be achieved by utilizing motion capture systems. Non-visual tracking systems, such as wearable sensors, or visual-based tracking systems, such as camera-based systems, can be implemented to automate stroke assessment. In the field of non-visual tracking systems, several sensor technologies such as inertial sensory systems (IMUs), ultrasonic localization systems (UMSs), electromagnetic measurement systems (EMSs), and glove-based wearable sensors are employed in the field of motion capturing [12,13]. The visual-based tracking system, which uses devices such as optical or camera-based sensors, is deployed. The camera-based techniques consist of a marker-based and markerless tracking system. The marker-based method can use optical-passive or optical-active procedures. The optical-passive procedures include those such as Vicon (Motion System Ltd., Oxford, UK), which uses an optical, infrared camera tracking the retroreflective markers. The optical-passive system utilizes LED markers that radiate light that specific cameras can trace. In other words, the optical-passive procedures only reflect the light coming towards the markers, in contrast to the optical-active procedures, which produce the light that is then gathered using camera-based techniques [13]. The Vicon system (Motion System Ltd., Oxford, UK), an optical passive, produces the 3D positions of objects by combing several cameras’ 2D positions, which are derived from the reflective markers placed on the body [13]. Several features, such as segment orientation, joint angle, and acceleration, can be derived from the Vicon camera system [13]. The wearable sensors or inertial measurement systems (IMSs), such as Xsens (Xsens MVN Awinda, Xsens Technologies, the Netherlands), use fused multiple inertial sensors, including a 3D gyroscope, 3D acceleration, and 3D magnetometer, to collect data such as position, linear and angular acceleration, velocity, etc. MEMS motion sensors are utilized to develop a sensor fusion structure [14]. There are several studies focused on using wearable sensors and camera-based systems applying machine learning technology for activity recognition [15,16,17,18,19,20,21,22,23] measurement classification [24,25,26,27,28,29,30,31,32,33,34], and clinical assessment simulation [35,36,37,38,39,40,41,42,43]. However, the main machine learning technique primarily uses supervised learning to automatically predict assessment scores. In clinical assessment simulations, a few studies have used unsupervised learning[1,44,45] to only determine outliers or homogeneous movements, then utilized regression classifiers to predict scores. No study has investigated severity levels using unsupervised learning and trunk displacement features in the frequency domain for post-stroke smart assessment. In this paper, the compensatory movements, or trunk displacement, have been used to label each cluster and then been compared with the ground truth FMA score achieved by clinician experts. The clustering of data using two different datasets (camera and wearable) in the frequency domain using position and linear acceleration features is an additional novelty in, and contribution made by, this paper.

The main contributions of the reported research are summarized below:

- The function of the affected hand in post-stroke patients (level of severity) was investigated using unsupervised learning.
- The general movements categorized as activities of daily living, such as holding a cup and drinking, eating apples, answering the phone, etc., were utilized.
- For the first time, position data in the frequency domain was used in addition to the acceleration data.
- The novel labeling method for each cluster using trunk displacement is one of the main contributions made by this study.
- In the study, the proposed method investigated not only wearable datasets but also camera-based datasets.

This paper is organized as follows. Section 2 describes the related works. Clustering analysis and consensus learning are discussed in Section 3 and Section 4, respectively. The proposed assessment model using consensus-based clustering is demonstrated in Section 5. The material and methods, preprocessing, and proposed labeling method are deliberated in Section 6. The data preprocessing is shown in Section 7. The experimental results are presented in Section 8. Furthermore, a discussion of the results is offered in Section 9. Finally, the conclusion and future are deliberated in Section 10.

## 2. Related-Work

Supervised learning predicts severity levels by developing various connections between patient attributes and the effects of interest, which have been investigated the most within the literature [46,47]. The studies developed on the automated assessment of the motor function of the upper extremity are divided into three types, including those on activity recognition [15,16,17,18,19,20,21,22,23], measurement classification [3,24,25,26,27,28,29,30,31,32,33,34], and clinical assessment simulation [1,35,36,37,38,39,40,41,42,43]. Additionally, the assessments have been utilized by employing various sensors. Wearable sensor-based systems are greatly used (such as IMUs or accelerometers [15,16,17,18,19,20,21,22,23], barometers [48,49], Flex sensors [21], EMG sensors [24,25], pressure sensors, etc.) [21,26,27,28,29,30,31,32], as are camera-based systems [5,33,34,35,36], to automate the stroke assessment test by reducing barriers and the obligations of experts or physiotherapists [1]. Wearable and camera-based sensors have been used for clinical treatment and rehabilitation [12,47].

### 2.1. Wearable Sensors

The authors in [1] chose 23 stroke patients with FMA-UE scores of less than 30, which is found in severe stroke patients, and used unsupervised learning to determine the homogeneous movement, outlier movements, and all moving components. A study by [26] used the Xsens wearable system and 17 sensors to define the correlation between FMA score and body movements while patients performed daily activities. Two IMU sensors were developed by the [27] group to assess the outcomes of rehabilitation treatments. However, these two studies did not implement machine learning techniques. Additionally, Ref. [28,29] estimated the functional ability scale using accelerometer sensors attached to the arm, upper arm, and hand. The Random Forest technique used the acceleration data to predict FMA scores. In addition to the accelerometer, flex sensors were attached to the body by the [21] study to monitor patient movements. The Extreme Learning Machine (ELM) was applied to predict FMA scores [21]. Researchers employed a rule-based classification [37] to estimate each patient’s FMA score using accelerometer sensors affixed to the upper arm and forearm. The summary of the work conducted using wearable sensors is presented in Table 1. In the literature, the assessment type has been investigated based on three categories: clinical emulation [1,19,20,21,22,23,30,38,39,40], movement classification [3,17,18,25,41,42,43,50,51,52,53,54], and activity recognition [15,16,24,44,48,49,55,56,57,58,59]. As this study focused on clinical emulation, Table 1 describes only the summary of this category. Several studies deployed individual accelerometers [19,20,22,37], and some studies combined IMUs with different sensors such as flex sensors [60,61]. While some studies used healthy participants [20,54,62], others used stroke patients as participants [1,22,23,38,39,40]. Support Vector regression (SVR) was the most used classifier [21,22,40]; this was primarily used in regression problems for clinical assessments where participants were given clinical scores [1,19,20,21,22,30,37,38,40]. A few researchers also employed the Random Forest (RF) technique [19,20,30]. In a few studies, the unsupervised machine learning method utilized the k-means [32] and DBScan [1] methods. The work in [32] investigated the Functional Ability Scale (FAS) assessment test using the K-means cluster and demonstrated the clustering results correlation to movement quality examine by the FAS. The authors in [45] used a hierarchical cluster to define the level of severity based on FMA scores. No study directly investigated the severity levels of strokes using unsupervised AI-driven models without available labeling.

### 2.2. Camera-Based Sensors

The summary of studies developed using the camera-based sensors is described in Table 2. The Kinect camera is one of the cameras used in motion capture research [63,64]. The Kinect camera is also combined with the Myo armband [9,65], force-sensing and resistor-sensing [5,35], pressure sensors or gloves sensors [34]. Different supervised machine learning techniques have been used, such as Artificial Neural Networks [33], SVM [34,66], and rule-based classification techniques [5,66]. The Vicon camera (Vicon Motion System Ltd., Oxford, UK) is also used to capture human motion, and the kinematics data can be derived using the Nexus software. However, no study has investigated severity levels of strokes using unsupervised learning and trunk displacement features in the frequency domain in post-stroke smart assessment. In this study, the compensatory movements or trunk displacements were used to label each cluster and then compared with the ground truth FMA scores achieved by clinician experts. The clustering of data using two different datasets (camera and wearable) in the frequency domain using position and linear acceleration features is an additional novelty in, and contribution made by, this paper.

## 3. Clustering Analysis

Different categories of clustering methods are based on their properties: for example, hard, soft, distance-based, and density-based clustering. Eight baseline clustering methods are employed: the Fuzzy C-means, K-means, the Self-Organizing Map (SOM), Gaussian Mixture Models, DBScan, and hierarchical, spectral, and OPTICS clustering.

### 3.1. K-Means Clustering

The K-means method [67] aims to minimize the sum of squared distances between each data point and the centroids of the assigned cluster, as defined in Equation (1).

(1) $$\arg\min_{C,M\;}\sum_{i=1}^{K}\sum_{j=1}^{N}||X_{i}-\mu_{j}||{}^{2}.{\left[C\right]}_{ij}$$

Here, *k* is the number of data points, N is the number of clusters, *C_ij_* is the *(i,j)*-th element of matrix *C* (it equals 0 if a data point does not belong to cluster *j*, and 1 otherwise), *μ_i_* is the centroid of the *j*^th^ cluster (i.e., it is the average of all data points assigned to cluster *j*), and $||X_{i}-\mu_{j}||{}^{2}$ is the Euclidean distance between two points.

### 3.2. Fuzzy C-Means Clustering

The cluster center “*c*” is randomly selected and the probability of cluster membership for an i^th^ data point to the j^th^ cluster is calculated thus [67]:

(2) $$\mu_{ij}=\frac{1}{\sum_{k=1}^{c}{\left(d_{ij}/d_{ik}\right)}^{\left(2/m-1\right)}}$$

*m* is the fuzziness parameter or factor, *c* denotes the cluster number, and *d_ij_* indicates the Euclidean distance between the *j^th^* cluster center and *i^th^* data point. $\mu_{ij}$ represents the degree of membership of the *i^th^* data to the j^th^ cluster center. After allocating data points to the *j^th^* cluster, the center of each cluster is defined thus:

(3) $$v_{j}=\frac{\sum_{i=1}^{n}\mu_{ij}^{m}\times x_{i}}{\sum_{i=1}^{n}\mu_{ij}^{m}}\;\forall j=1,2,\ldots .c$$

This iteration continues until the following equation is minimal:

(4) $$J\left(U,V\right)=\sum_{i=1}^{n}\sum_{j=1}^{c}\mu_{ij}^{m}||x_{i}-v_{j}||{}^{2}$$

### 3.3. SOM Clustering

Let *X = {x1, x2, …, xn}* be the set of input vectors and *W = {w1, w2, …, wm}* be the set of weight vectors for the nodes in the grid. The SOM clustering tries to minimize the objective function *J(W)* (Equation (5)) and update the weights of the nodes in the grid (Equation (6)):

(5) $$J\left(W\right)=\sum_{i}||x_{i}-w_{i}||{}^{2}$$

(6) $$w_{i\left(t+1\right)}=w_{i}\left(t\right)+\eta \left(t\right)\times h\left(c_{i},\;t\right)\times \left(x_{i}-w_{i}\left(t\right)\right)$$

Here, *t* is the iteration number, *η(t)* is the learning rate at iteration *t*, and *h(c_i_, t)* is the neighbourhood function that determines the influence of the input vector *x_i_* on the weight vector *w_i_* based on the distance between *c_i_* and the winning node, and *c_i_* is the index of the winning node in the grid for the input vector *x_i_* [68,69,70].

### 3.4. Hierarchical Clustering

In hierarchical clustering, the relationship between each cluster is based on hierarchy, and a dendrogram is the output of the included clusters. Initially, this process considers the full dataset as a cluster and then the hierarchical levelling is developed based on a distance matrix. The distance between each pair of data points is calculated and then the closest pair is selected and merged. This process is repeated iteratively until all clusters have been merged/split. The objective of optimization in hierarchical clustering is to find the optimal hierarchy of nested clusters that best represents the underlying structure of the data. The quality of the clustering solution can be evaluated using external or internal validation metrics such as the adjusted Rand index, the silhouette score, or the cophenetic correlation coefficient [71].

### 3.5. Spectral Clustering:

Spectral clustering is a graph partitioning problem that identifies node neighbourhoods and the edges connecting them based on graph theory. This method involves transforming the data into a new representation using the eigenvalues and eigenvectors of a matrix derived from the data. The process begins by constructing a similarity graph or matrix that captures the pairwise similarity or dissimilarity among each pair of data points. The graph can be constructed in different ways depending on the problem. Still, the most common approach is to use a Gaussian kernel function to measure the similarity between two data points based on their distance. Once the similarity matrix is constructed, the eigenvectors and eigenvalues of the matrix are computed using techniques from linear algebra. The *k* eigenvectors corresponding to the *k* smallest eigenvalues are then used to represent the data in a lower-dimensional space. This is conducted by treating the eigenvectors as new coordinates for the data points. Spectral clustering can be computationally expensive and requires the choice of several parameters such as the number of clusters and the similarity measure [72].

### 3.6. Gaussian Mixture Models Clustering:

Gaussian Mixture Models (GMM) clustering is a soft clustering method based on probability density assessments that applies the Expectation-Maximization algorithm and creates ellipsoid-shaped clusters. A GMM is composed of several Gaussian distributions and has mean and center. In addition, the covariance is also defined for the GMM clustering method. The mixing probability, which defines the Gaussian function size, is also determined for GMM clustering, and this enhances the ability of this method to deliver a numerical quantity of capability per total of clusters when compared to hard clustering methods such as the K-means method. Given a dataset *X = {x₁, x₂, ..., x_N_}* and a GMM model with *K* Gaussian components, the goal is to find the optimal values of the parameters *θ = {w₁, ..., w_K_, μ₁, ..., μ_K_, Σ₁, ..., Σ_K_}*, where *wᵢ* is the weight of the *i*-th component, *μᵢ* is its mean vector, and *Σᵢ* is its covariance matrix. The optimization problem aims to maximize the log-likelihood of the observed data as depicted below:

(7) $$LL\;\left(\theta \right)=\sum_{i}\log[\sum_{k}w_{k}N\left(x_{i}|\mu_{k},\sum^{\;}k)\right]$$

Here, $N\left(x_{i}|\mu_{k},\sum^{\;}k\right)$ is the Gaussian probability density function with mean *μₖ* and covariance matrix $\sum^{\;}k$ evaluated at data point *xᵢ*. The Expectation-Maximization (EM) algorithm is used to find the optimal values of these parameters. The algorithm alternates between the E-step and the M-step. In the E-step, the posterior probabilities *γᵢₖ* of each data point *xᵢ* belonging to each component *k* are computed thus:

(8) $$\gamma_{ik}=\frac{w_{k}N\;\left(x_{i}|\mu_{k},\sum^{\;}k\right)}{\sum_{1}w_{1}N\left(x_{i}|\mu_{1},\sum^{\;}1\right)}$$

Here, $\underset{1}{\sum }w_{1}N\left(x_{i}|\mu_{1},\sum^{\;}1\right)$ is the total probability of data point *i* across all components.

In the M-step, the parameters are updated as follows:

(9) $$w_{k}=\sum_{i}\frac{\gamma_{ik}}{N}$$

(10) $$\mu_{k}=\frac{\sum_{i}\gamma_{ik}x_{i}}{\sum_{i}\gamma_{ik}}$$

(11) $${\sum^{\;}}_{k}=\frac{\sum_{i}\gamma_{ik}\left(x_{i}-\mu_{k}\right){\left(x_{i}-\mu_{k}\right)}^{T}}{\sum_{i}\gamma_{ik}}$$

where N is the total number of data points. The EM algorithm iteratively updates the parameters until convergence is reached. The final set of parameters represents the optimal solution to the GMM clustering problem [73,74].

### 3.7. DBScan Clustering:

The DBSCAN (Density-Based Spatial Clustering of Applications with Noise) algorithm does not require specifying the number of clusters in advance, and can handle clusters of arbitrary shape. DBSCAN defines two parameters: the minimum number of points (MinPts) required to form a dense region and a distance threshold (eps) that determines the size of the neighborhood around each data point. Let D be the dataset of n data points *{x_1_, x_2_, ..., x_n_}* and let eps and MinPts be the distance threshold and minimum number of points, respectively. The DBSCAN algorithm optimizes the clustering by finding the following sets of points:

1. Core points: A point *x* in *D* is a core point if it has at least MinPts points in its eps-neighborhood, including itself.
2. Border points: A point *y* in *D* is a border point if it is not a core point but has at least one core point within its eps-neighborhood.
3. Noise points: A point *z* in *D* is a noise point if it is neither a core nor a border point.

The DBSCAN algorithm groups the core points and their border points into clusters. Two core points belong to the same cluster if they are directly or indirectly reachable from each other through a series of core points. DBSCAN tries to maximize the number of points assigned to a cluster while minimizing the number of noise points. DBSCAN adjusts the parameters eps and MinPts to find the optimal clustering [1,75].

### 3.8. OPTICS Clustering:

OPTICS, which stands for ‘ordering points to identify cluster structure’, is another density-based clustering method similar to DBScan. However, the reachability distance plot in OPTICS is an advancement over that in DBScan. The reachability distance between *p* and *q* is the smallest distance from *p* if *p* is the core object. OPTICS uses a priority queue data structure to efficiently order the points based on their densities and avoid the computation of pairwise distances between all points, which can be computationally expensive. Instead, the priority queue allows the algorithm to consider only the points relevant to the current point being processed. Another optimization used in OPTICS clustering is a data structure called a core distance tree, which stores the core distances of all points in the dataset. The core distance of a point is the minimum distance at which a point can be considered part of a dense region. The core distance tree efficiently computes the reachability distances between points in the dataset [76].

## 4. The Consensus Solvers

Consensus clustering combines multiple clusterings of the same data set into a single-consensus clustering solution to obtain a more stable and robust clustering solution that captures the common structure across different clusterings.

### 4.1. Meta-Clustering Algorithm (MCLA) Consensus Solver

The Meta-Clustering Algorithm (MCLA) is centered around clustering clusters and provides confidence estimates for object membership within clusters. The performance of MCLA depends on the choice of clustering algorithms, the combination method, and the quality of the individual clusterings. Its first goal is to transform the provided cluster label vectors into a hypergraph representation appropriate for subsequent analysis. Any set of clusterings can be mapped to a hypergraph composed of vertices and hyperedges. The hyperedge is a matrix of binary membership indicators. Each column represents a cluster, and the rows corresponding to objects with unknown labels are populated with zeros in the indicator matrix while 1 denotes an object with a known label. As a result, each cluster can be mapped to a hyperedge, and the set of clusterings can be represented as a hypergraph. For the MCLA, clusters are represented as hyperedges. The MCLA aims to group and merge related hyperedges, assigning objects to the resulting collapsed hyperedge based on their strongest participation. A graph-based clustering approach achieves the determination of related hyperedges for collapsing.

### 4.2. HyperGraph Partitioning Algorithm (HGPA) Consensus Solver

The HyperGraph Partitioning Algorithm (HGPA) directly repartitions the data by leveraging the existing clusters as indicators of strong associations. In addition, it can be expressed as partitioning a hypergraph by removing the smallest number of hyperedges. In the HGPA, the objective is to partition the hypergraph into a set *of l* disjoint subgraphs (called partitions) such that some criterion is optimized. The HGPA works by constructing a weighted hypergraph where each vertex represents a hyperedge and the weight of each vertex is proportional to the cost of assigning it to a specific partition. [77].

### 4.3. Cluster-based Similarity Partitioning Algorithm (CSPA) Consensus Solver

The CSPA partitions a given dataset into distinct clusters by analyzing the similarities between the objects in the dataset. The algorithm operates by building a similarity matrix that evaluates the similarities between pairs of objects in the dataset. The choice of similarity measure can be any distance metric or appropriate similarity measure for the data. After constructing the similarity matrix, the algorithm initially groups clusters by clustering objects that display high pairwise similarities. Subsequently, this initial set of clusters is improved using a hierarchical clustering algorithm that iteratively combines pairs of clusters with high similarities until the desired number of clusters is obtained. When merging the clusters, the algorithm leverages a cluster similarity measure to determine which pairs of clusters should be combined in situations where the weights are proportionate to the sizes of the clusters [77].

### 4.4. Hybrid Bipartite Graph Formulation (HBGF) Consensus Solver

**Figure 1:**
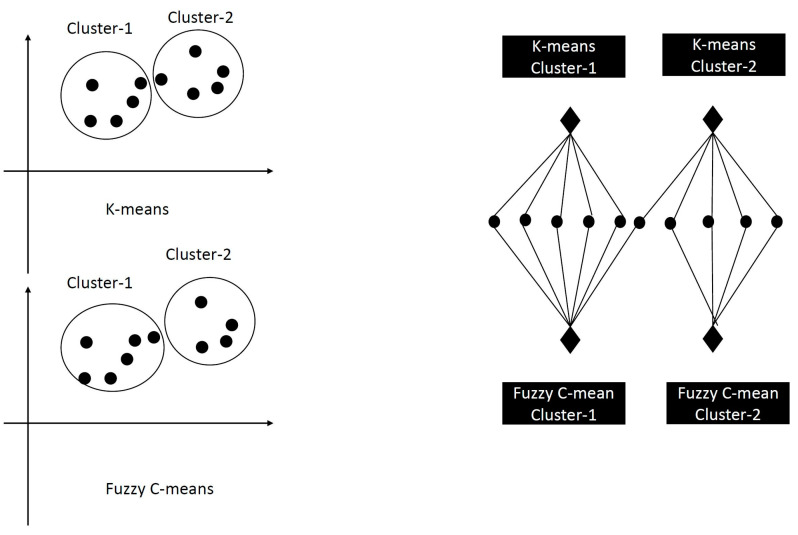
The HBGF consensus clustering bipartite graph.

The Hybrid Bipartite Graph Formulation (HBGF) creates a bipartite graph of vertices and edges. An edge will only connect an instance vertex to a cluster vertex if that instance belongs to that cluster. New cluster vertices are added if a fresh clustering is incorporated into the ensemble, with these vertices linked to the instances they contain. Round vertices and clusters represent the instances illustrated by diamond-shaped vertices. The graph’s edges each possess a single weight, with any edges possessing zero weights being excluded. The bipartite graph is partitioned into non-overlapping clusters using a partitioning algorithm such as spectral clustering or the k-means method. The goal of the partitioning algorithm is to minimize the number of cut edges between clusters while balancing the weights of the vertices [78]. Figure 1 presents the HBGF consensus clustering using two individual clusterings: K-means and Fuzzy C-means clustering.

## 5. The Proposed Post-Stroke Severity Assessment Model using Modified NMF-Consensus Solver (PSA-MNMF)

The proposed consensus clustering algorithm, PSA-NMF, combines various clusterings into one united clustering, i.e., creating cluster consensus, to produce more stable and robust results compared to any individual clustering method. We developed a modified nonnegative matrix factorization [79] (MNMF) as an enhanced consensus solver by factorizing the consensus matrix into two nonnegative matrices that represent the underlying structure of the data. In our algorithm, once the MNMF phase is completed, an exhaustive search is executed to find the best optimal combinations of the consensus toward robust results. Each of these methodologies is described next.

### 5.1. The Modified Nonnegative Matric Factorization (MNMF) Consensus Solver

Assume *A* defines the data point *A = {a_1_,…, a_n_}*, which contains n data points. Then, the partitions *B* of data point A is *B = {b^1^, b^2^, …., b^E^}*, *E = 1,…., E,* and the set of clustering in each partition is *D_t_ = {d_1_^f^, d_2_^f^,…. d_m_^f^}*. m denotes the number of clusters for the partition *B^T^*. The number of clusters m can differ for each cluster and $A=\cup_{h=1}^{m}D_{h}^{t}$. Now, the distance between two partitions *B^1^* and *B^2^* can be defined based on the MNMF solver as follows:

(12) $$f\left(b^{1},b^{2}\right)=\sum_{i,j=1}^{n}f_{i,j}\left(b^{1},\;b^{2}\right)$$

For each element,

(13) $$f_{i,j}\left(b^{1},\;b^{2}\right)=\{\begin{array}{c} 1\;\left(i,j\right)\notin \;D_{k}\left(b^{1}\right)\;and\;\left(i,j\right)\in \;D\left(b^{2}\right)\\ \;1\;\left(i,j\right)\in \;D_{k}\left(b^{1}\right)\;and\;\left(i,j\right)\notin \;D_{k}\left(b^{2}\right)\\ 0\;non\;of\;the\;above \end{array}$$

where *i* and *j* are members of different clusters in partition *b^1^*, denoted thus: $\left(i,j\right)\notin \;D_{k}\left(B^{1}\right)$. Then, one approach can be to define the connectivity matrix as follows:

(14) $$CM_{ij}\left(b^{t}\right)=\{\begin{array}{c} 1\;\left(i,j\right)\in \;D_{k}\left(b^{t}\right)\\ 0\;\left(i,j\right)\notin \;D_{k}\left(b^{t}\right)\; \end{array}$$

The *f_ij_* (*b^1^*,*b^2^*) values are defined according to the connectivity matrix as follows:

(15) $$f_{ij}\left(b^{1},b^{2}\right)=\left|CM_{ij}\left(b^{1}\right)-CM_{ij}\left(b^{2}\right)\right|={\left|CM_{ij}\left(b^{1}\right)-CM_{ij}\left(b^{2}\right)\right|}^{2}$$

The *f_ij_* (*b^1^*,*b^2^*) value is 0 or 1.The consensus clustering b* can be derived thus:

(16) $$\underset{b^{*}}{\min}g=\frac{1}{T}\sum_{t=1}^{T}f_{ij}\left(b^{t},b^{*}\right)=\frac{1}{T}\sum_{t=1}^{T}\sum_{k=1}^{n}{\left[CM_{ik}\left(b^{t}\right)-CM_{ik}\left(b^{*}\right)\right]}^{2}$$

Assuming that the solution for the optimization equation above is U_ik_ = CM_ik_ (B*), the average consensus association between *i* and *k* is as follows:

(17) $${\tilde{CM}}_{ik}=\frac{1}{T}\sum_{t=1}^{T}CM_{ik}\left(b^{t}\right)$$

The average squared difference from the consensus association is as follows:

(18) $$\tilde{CM}:\;\Delta CM^{2}=\frac{1}{T}\sum_{t}\sum_{i,k}{\left[CM_{ik}\left(b^{t}\right)-\tilde{CM_{ik}}\right]}^{2}$$

The smaller $\Delta CM^{2}$ is, the closer to each other the partitions are. The $\Delta CM^{2}$ is constant, and therefore,

(19) $$G=\frac{1}{T}\;\underset{t}{\sum }\underset{ik}{\sum }{\left(CM_{ik}\left(b^{t}\right)-{\tilde{CM}}_{ik}+{\tilde{CM}}_{ik}-U_{ik}\right)}^{2}\;=\Delta CM^{2}+\underset{i,k}{\sum }{\left({\tilde{CM}}_{ik}-U_{ik}\right)}^{2}=\tilde{CM}-U^{2}\;$$

Then, the optimization problem for consensus clustering is expressed as follows:

(20) $$\underset{\tilde{{\tilde{H}}^{T}\;\tilde{H}=1,\;\tilde{H}\geq 0}}{\min}{\tilde{||CM-\tilde{N}\;{\tilde{N}}^{2}}||}^{2}$$

This norm is called the Frobenius norm. This equation indicates that the consensus association clustering used is consensus clustering. Now, the next task is to define an NMF clustering solver. The nonnegative matrix factorization (NMF) solver uses nonnegative data (*A*) factorized to two nonnegative matrices like *P* and *Q (A = PQ*). For the NMF formulation, the clustering indicator of a matrix *N = {0,1}^nxk^* can be indicated as a clustering solution, and by definition the constraint for this matrix is that in each row, only one “1” can exist and the rest of the elements must contain zeros.

U_ik_ = NN^T^ or U_ik_ = (NN^T^)_ik_ (21)

If i and k belong to the same cluster since the dot product is the multiplication of *i* by *k*, the results will equal 1 *(NN^T^)_ik_ = 1*; otherwise, *(NN^T^)_ik_ = 0*. With the constraints of *U = NN^T^*, the consensus clustering is optimized as follows:

(22) $$\underset{N}{\min}||\tilde{CM}-NN^{T}||{}^{2}$$

Let us assume that *(N^T^N)_jl_ = 0* when j is not equal to l and *i* and *j* do not belong to each other. Additionally, we must consider that *(N^T^N)_jj_ = |C_j_| = r_j_*, where *C_j_* denotes cluster *j*, and that in the matrix there will be only non-zero r*_j_* values. Let *L = diag(N^T^N) = diag (r_1_, r_2_, ….r_k_)* where *N^T^N = L*. Then, the optimization problem for this case can be written as follows:

(23) $$\underset{NN^{T}=1,\;L,\;N\geq 0}{\min}||\tilde{CM}-NN^{T}||{}^{2}$$

This optimization can be easier to solve compared to Equation (21). However, the cluster size should be defined. Therefore, the cluster size should be eliminated.

(24) $$\tilde{N}=N{\left(N^{T}N\right)}^{\frac{-1}{2}}$$

(25) $$NN^{T}=\tilde{N}L{\tilde{N}}^{T},\;{\tilde{N}}^{T}N=N\left(N^{T}N\right){\;}^{-1}N=1$$

Then, the optimization equation is as depicted:

(26) $$\underset{{\tilde{N}}^{T}\tilde{N}=1,\;L\tilde{,\;N}\geq 0}{\min}||\tilde{CM}-\tilde{N}L{\tilde{N}}^{T}||{}^{2}$$

${\tilde{N}}^{T}$ and *L* are both acquired as solutions for this problem. This formulation demonstrates that consensus clustering can be defined as a symmetric nonnegative matrix factorization. The optimization equation of the MNMF is as follows:

(27) $$\underset{N,\;L\geq 0}{\min}||\tilde{CM}-\tilde{N}L{\tilde{N}}^{T}||{}^{2}$$

Given *N^T^N=1*, for each iteration, the procedure is updated as follows:

(28) $$N_{oi}\;\leftarrow \;N_{oi}\;\sqrt{\frac{{\left(CM\;N\;L\right)}_{oi}}{{\left(NN^{T}CM\;NL\right)}_{oi}}}\;$$

(29) $$L_{ie}\;\leftarrow \;L_{ie}\;\sqrt{\frac{{\left(N^{T}CM\;N\right)}_{ie}}{{\left(N^{T}N\;L\;N^{T}N\right)}_{ie}}}\;$$

In conclusion, the above explains the formulation of an algorithm of [78]. *L* was restricted to being a diagonal matrix, but, in this equation, *Y* is not restricted to being a diagonal matrix. However, at some point, it can change to being a diagonal matrix; in that case, it will remain a diagonal matrix.

### 5.2. Exhaustive Search

An exhaustive search phase was conducted in this study to find every potential solution in order to obtain a desirable solution. We tried to find the best performance among a combination of baseline clustering methods within the solver consensus by attempting every possible combination. The model ran 100 times to decrease the variance; the best 10 combinations were reported each time. The 10 best performances (quantified by selecting a proper assessment measure, such as F-score) were reported. The final best combination that appeared the most was selected to be reported in the results.

### 5.3. THE PSA-MNMF Consensus Clustering Algorithm

**Figure 2:**
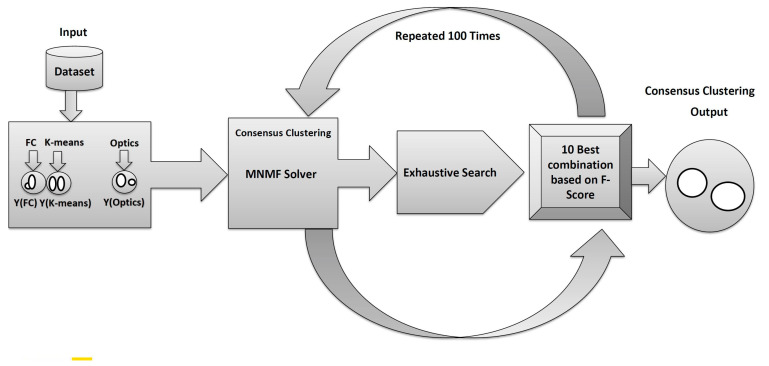
The proposed PSSA-MNMF consensus clustering method is demonstrated.

The input data set is provided in the PSA-MNMF consensus clustering algorithm and a set of clustering algorithms in order to generate the individual clusterings. Next, the baseline clustering algorithms are applied to the input data set to generate a set of individual clusterings. Each clustering algorithm may produce a different result due to its unique assumptions, parameters, and randomness. A consensus matrix is constructed based on the scores of similarity between the individual clustering. The consensus matrix is a square matrix representing the degree of agreement between each pair of data points in the input data set. The consensus matrix is then factorized into two nonnegative matrices, *W* and *H*, using the MNMF. The factorization can be expressed as C ≈W*H, where C is the consensus matrix, W is the cluster assignment matrix, and H is the consensus centroid matrix. The rows of the consensus centroid matrix are used as input for a chosen clustering algorithm to obtain a final consensus clustering solution. The clustering algorithm choice may differ from the individual baseline clustering algorithms. Finally, each data point is assigned to the corresponding consensus cluster. Algorithm 1 summarizes the proposed PSA-MNMF algorithm and Figure 2 describes the consensus part of the proposed method.


**Algorithm 1: PSA-MNMF**
**Input**: Dataset *A={a1,…., an}*, a set of partitions B of data points B = {b1,b2, ….., bt} such that each partition B consists of a set of clustering *Dt= {d_1_^t^, d_2_^t^, …., d_k_^t^}* that uses a selected clustering methodology. **Output:** The set *H* of *B* heterogeneous clusterings that included the 10 best and highest F-scores (or performance metrics α) and appeared in all 100 runs when using the exhaustive search method.  **Initialization:** Calculate the *X*-cluster= {The results of each clustering Initialize *H*= {}. Define the connectivity matric CM as follows:            $CM_{ij}\left(b^{t}\right)=\{\begin{array}{c} 1\;\left(i,j\right)\in \;D_{k}\left(b^{t}\right)\\ 0\;\left(i,j\right)\notin \;D_{k}\left(b^{t}\right)\; \end{array}$
Define a matrix *N_ixk_* such that in each row only “1” can exist and the rest of the values should be zeros. Calculate the *NN^T^*. If i belongs to k, the results will equal 1, otherwise they will equal zero. Define *L* as *L = N^T^N*.  **Begin** $\mathrm{Step}\;1\text{:}\;\mathrm{The}\;\mathrm{optimization}\;\mathrm{equation}\;\mathrm{for}\;\mathrm{PSA}-\mathrm{NMF}\;\mathrm{is}\;\mathrm{calculated}\;\mathrm{using}\;\mathrm{the}\;\mathrm{equation}\text{:}\;\underset{N,\;L\geq 0}{\min}||\tilde{CM}-\tilde{N}L{\tilde{N}}^{T}||{}^{2}$, where *N^T^N=1*
$\mathrm{Step}\;2\text{:}\;\mathrm{At}\;\mathrm{each}\;\mathrm{iteration},\;\mathrm{the}\;N\;\mathrm{value}\;\mathrm{will}\;\mathrm{be}\;\mathrm{updated}\;\mathrm{using}\;\mathrm{this}\;\mathrm{equation}\text{:}\;N_{oi}\;\leftarrow \;N_{oi}\;\sqrt{\frac{{\left(CM\;N\;L\right)}_{oi}}{{\left(NN^{T}CM\;NL\right)}_{oi}}}$
$\mathrm{Step}\;3\text{:}\;\mathrm{At}\;\mathrm{each}\;\mathrm{iteration},\;\mathrm{the}\;L\;\mathrm{is}\;\mathrm{updated}\text{:}\;L_{ie}\;\leftarrow \;L_{ie}\;\sqrt{\frac{{\left(N^{T}CM\;N\right)}_{ie}}{{\left(N^{T}N\;L\;N^{T}N\right)}_{ie}}}$ **Step 5:** The exhaustive method finds the best performance metric from among the top 10 recorded combinations. **Step 4:** Steps 1-4 are repeated 100 times. **Step 6:** The final consensus clustering solution assigns each data point in the input data set to a consensus cluster. **Step 7:** The algorithm returns *H* and performance metrics α (including F-score, accuracy, precision, and recall) **End**

The proposed MNMF-based consensus clustering has several advantages over traditional consensus clustering methods. The MNMF can handle high-dimensional data sets and extract meaningful features that capture common structures across different clusterings. Moreover, the MNMF can provide a more interpretable clustering solution as it explicitly separates the cluster assignments from the consensus centroids. The PSA-MNMF is the first contribution toward post-stroke severity assessment that provides robust results using the position data and acceleration data in the frequency domain.

## 6. Data, Materials, and Methods

Generally, post-stroke motion datasets are rarely considered when choosing an open-source dataset. The U-limb datasets published in 2021 consist of 65 post-stroke and 91 healthy subjects collected in different clinical settings using the same protocol [80]. In this study, to deploy an unsupervised learning method, the data collected from stroke patients by utilizing wearable sensors and camera-based sensors were implemented from the U-limb datasets. Group research at the University of Zurich (UZH) [81] implemented 17 IMU sensor systems, collected using the Xsens suite (Awinda, Xsens Technologies B.V., Enschede, The Netherlands), for 20 stroke patients. IMUs included a 3D angular magnetometer, 3D accelerometers, and a 3D gyroscope. Table 3 describes the participants’ characteristics. The FMA-UE score for the patients, which was 46.00 ± 10.16, was in the moderate and mild categories according to the study [45]. Both affected hands and non-affected hands were used for this study. The dataset selected for this research is an open-source dataset. The mean age of the participants in this study was 61.00 ± 10.69, including 5 females and 15 males. 11 right hands and 9 left hands were affected, and it was known that there was only one person with a dominant left hand. The four grasping-action activities chosen for this research are described in Table 3. The camera-based dataset was dataset1 and the sensor-based dataset was dataset2.

The Hannover Medical School (MHH) research group collected position data on healthy and stroke-patient participants deploying motion capture technologies. This system comprised 12 MX Vicon cameras (Vicon Motion System Ltd., Oxford, UK) operated by Version 1.8.5 of the Nexus software. There were 21 passive markers attached to the upper body (thorax, upper arm, and forearm) to capture arm movements. The number of stroke patients attending this research was 20—12 male and 6 female—and the mean age of the participants was 49.88 ± 16.92 years. The FMA-UE for this group was 17.75 ± 2.05; since it was less than 29, it was considered to fall within the severe category [45]. The study captured only the affected hand of each stroke patient. There were 20 healthy participants included in this research, and 12 of them were male. The mean average age of the healthy group was 46.77 ± 15.25 years. The dominant hand was selected for testing in healthy participants, and 2 of the participants were left-handed. Each participant repeated the four tasks three times: the same as in the sensor data. This research group was selected for our study because the same experiment and research protocol had been employed with the UZH group. The Table 4 presents the characteristics of both wearable and camera-based system.

## 7. Data Preprocessing

**Figure 3:**
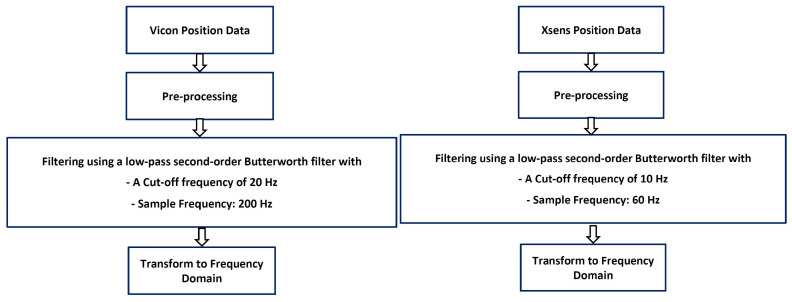
The preprocessing procedure.

The camera-based position data were collected at a 200 Hz sample rate. The position time series of camera data was filtered using a low-pass second-order Butterworth filter with a cut-off frequency of 20 Hz to lessen the high frequencies that were noise elements not created by humans. The wearable sensor data were collected with a sampling frequency of 60 Hz. The low-pass second-order Butterworth filter with a cut-off frequency of 10 Hz was applied. This section separately describes the wearable sensor dataset (dataset-1) and camera-based dataset (dataset-2). Figure 3 describes the general preprocessing steps to get the position data in frequency domains for the camera system and wearable sensor datasets.

### 7.1. Wearable Sensors (Dataset 1)

The 3D positions *(x, y, z)* of five major upper limb parts consisting of the hand, shoulder, upper arm, forearm, and sternum (T8) were selected for this research. Therefore, 5 features with 3D predictor variables (i.e., 15 features) were used for each side of the body. From the relevant equation, the linear acceleration data was derived from the position data. The position data and acceleration data were tested in the frequency domain. The following formula (in Equation 30) was used according to an earlier study [83] to decompose 3D to 1D each time and be independent of the orientations of sensors. Then, the mean value was derived for the acceleration data. In the equation below, *X, Y,* and *Z* are the three dimensions of acceleration in each step.

(30) $$Acceleration\;at\;each\;steps=\sqrt{X^{2}+Y^{2}+Z^{2}}$$

### 7.2. Camera-Based Sensors (Dataset-2)

The 3D positions from 11 markers at the wrist, ulnar bone, humerus bone, scapula, and trunk were collected from camera-based datasets. Nine markers were used for feature selection with 3 dimensions *(x,y,z)*. Therefore, 27 features were used for the camera dataset. The 4 markers on the trunk were used to define the trunk displacements. Similar to what had been done in the wearable sensor dataset, the acceleration was derived from the position data using a formula. The linear acceleration in the frequency domain and position data in the frequency domain were tested.

### 7.3. Trunk Displacement Measurement

Measurements were done according to an earlier study [84] using T8 from the wearable sensors and an average of 4 sensors on the trunk from the camera-based sensors. The measurements were done according to [84]. Trunk displacements were specified by differences in the position and orientation of the sensor located at the sternum [85]. The mean of the first 10 data points was subtracted from the position data in each step for all *x, y,* and *z* directions. The following equation was used to find one value for each step.

(31) $$Trunk\;Displacements=\sqrt{TD_{x}+TD_{y}+TD_{Z}}$$

Here, *TD_X_* is the trunk displacement in the *x* direction (or front) at each step, and TD_y_ and *TD_z_* are the trunk displacements in y and z, respectively. Based on the literature [85,86,87,88], trunk movements are called compensatory movements in stroke patients while performing tasks. The labelling for each cluster was assigned according to trunk displacement. For the camera-base dataset, 4 markers located at the trunk were selected, and for each marker, the displacement was calculated according to the above-mentioned method. Then, the average of these 4 markers was selected for the final trunk displacement to label each cluster.

### 7.4. Data Labeling

For stroke patients, extreme trunk displacement is a common motor compensation [89]. Accordingly, stroke survivors use trunk displacement as a compensatory movement for the activities of daily living [82,90]. Therefore, in a novel method for labelling, the trunk displacement was used to label each cluster. Accordingly, the more displacement was, the more severe the stroke level was. The lowest displacement average in each cluster was selected as the healthiest or mild level. The labelling method using trunk displacement is a novel one proposed in this paper. Each labelling result derived from each clustering was compared with the ground truth FMA score for each patient.

**Figure 4:**
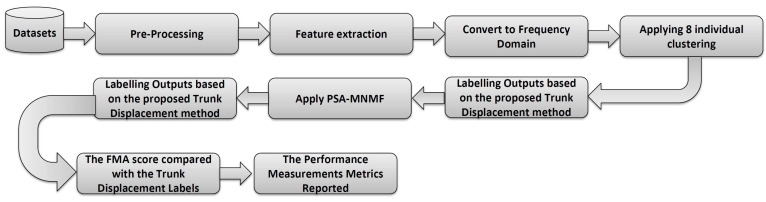
The step-by-step method for processing acceleration data, and implementing PSSA-MNMF consensus clustering, is demonstrated.

The visualization summary of step-by-step methods employed in this paper is presented in Figure 4.

## 8. Experimental Analysis and Results

This section provides the experimental results and analysis of the proposed PSA-MNMF algorithm as well as of baseline individual and consensus methods. Eight baseline clustering methods were employed: Fuzzy C-means clustering, K-means clustering, a Self-Organizing Map (SOM), Gaussian Mixture Models, DBScan, and Hierarchical, Spectral, and OPTICS clustering. The results of the consensus MCLA solver are also reported for making comparisons to the proposed PSA-MNMF. The accuracy, recall, precision, and F-score data are also reported. In this section, we have used two datasets, the wearable sensor-based dataset-1 and camera-based dataset-2. We have investigated the clustering results using a combination of position and acceleration in frequency. In this paper, for the number of clusters *k, k = 2* denotes ‘severe’ and ‘non-severe’, and *k* = 3 denotes ‘severe,’ ‘mild,’ and ‘non-severe.’

### 8.1. The Averaged Normalized Mutual Information (ANMI)

**Figure 5:**
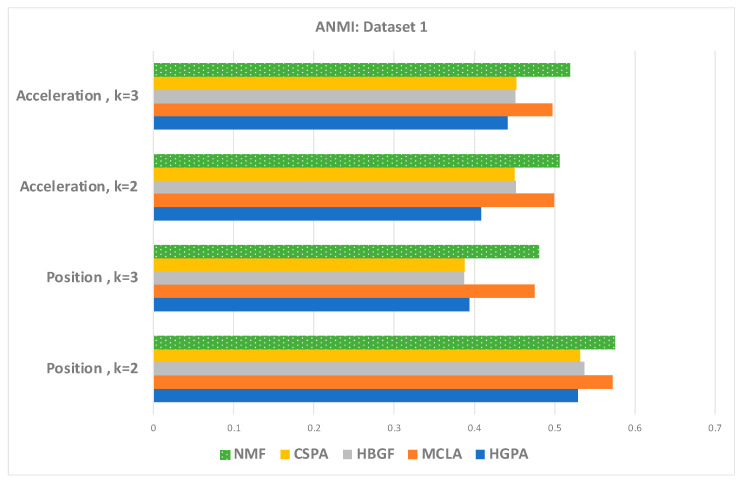
The ANMI: position and acceleration in the frequency domain (Dataset-1) for k = 2 and k = 3.

**Figure 6:**
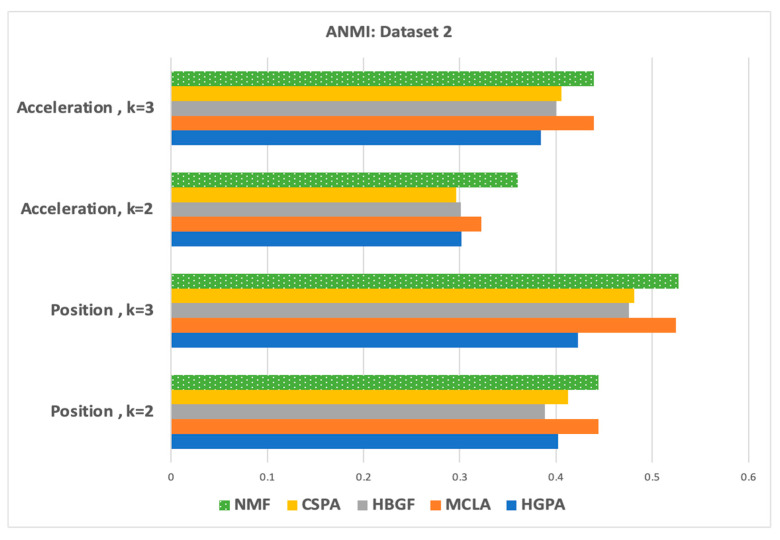
The ANMI: position and acceleration in the frequency domain (dataset-2) for k = 2 and k = 3.

If no prior information is available regarding the relative importance of the individual groupings, an appropriate objective for the consensus answer would be to identify a clustering that maximizes the information shared with the original clustering. Thus, to justify the choice of the NMF as a baseline solver for our proposed model, we have conducted a comparative analysis using Averaged Normalized Mutual Information (ANMI) to compare the five consensus clusterings including HGPA, MCLA, HBGF, CSPA, and NMF [77,91,92]. Mutual information, a symmetric measure that quantifies the statistical information shared between two distributions [93], serves as a reliable indicator of the information shared between a pair of clusterings. Averaged normalized mutual information measures the amount of information that two splits (clusters and class labels) share, regardless of the number of clusters. The Mutual Information score quantifies the degree to which these splits are correlated, indicating how much information can be inferred about one of them if the other is known. As the number grows higher and closer to 1, since it is normalized, the consensus clustering results show better performance. The ANMI results of the NMF, MCLA, CSPA, HGPA, and HGBF clusterings are reported in Figure 5 and Figure 6 for dataset-1 and dataset-2, respectively. The best baseline consensus solution for dataset-1 and dataset-2 was the NMF solver. Thus, the NMF solver method was selected as the base consensus model of our proposed model, with modified objective factors, as shown in Section 5.

#### 8.1.1. Performance Evaluation: Wearable Sensors (Dataset-1)

For *k* = 2 and *k* = 3, each algorithm ran 100 times to reduce the variance of the results. For k = 2, the computational time was about 0.38 s for each clustering run and approximately 95.35 s for the consensus clustering run. For *k* = 3, it was 0.215 s for each clustering run and approximately 82.94 s for the consensus clustering run. The cut-off frequency for the filter was assigned to be 10 Hz for *k* = 2 and *k* = 3. Table 5, Table 6 and Table 7 present the results of the position data on wearable position and acceleration and the merged data on acceleration and position in the frequency domain for dataset-1, respectively, for *k* = 2. Similarly, the results for k = 3 are shown in Table 8, Table 9 and Table 10. In Table 5 and Table 8, the performance results of the position data of wearable sensors in the frequency domain are presented. Table 6 and Table 9 present the results of the acceleration data on the wearable sensors in the frequency domain. Table 7 and Table 10 show the merged data on acceleration in frequency and position in the frequency domain of the wearable sensor data.

**Figure 7:**
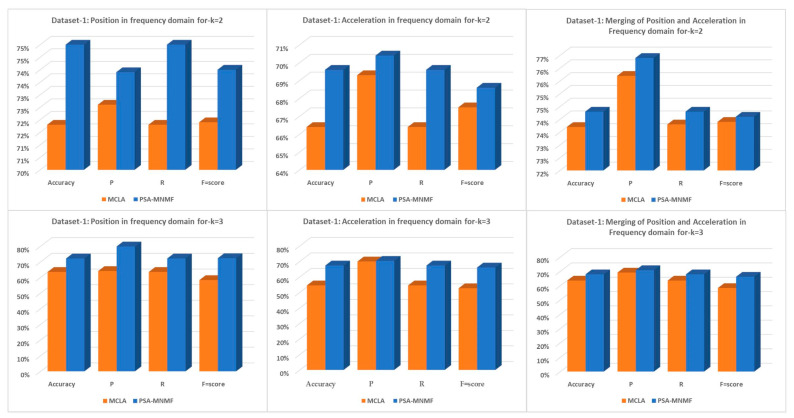
Dateset-1: PSA-MNMF vs. MCLA for k = 2 and k = 3.

As shown in Table 5, Table 6, Table 7, Table 8, Table 9 and Table 10, using a two-level and three-level assessment, the proposed PSA-MNMF showed the best performance compared to the baseline clustering methods. Figure 7 demonstrates the comparisons of position, acceleration, and merging of position and acceleration in frequency domains between the proposed PSA-MNMF and the MCLA consensus methods for k = 2 and k = 3. We can note that the proposed PSA-MNMF outperformed the MCLA by achieving higher accuracy, precision, recall, and F-score, making it suitable for clinical settings.

#### 8.1.2. Performance Evaluation: Camera-Based Data (Dataset 2)

**Figure 8:**
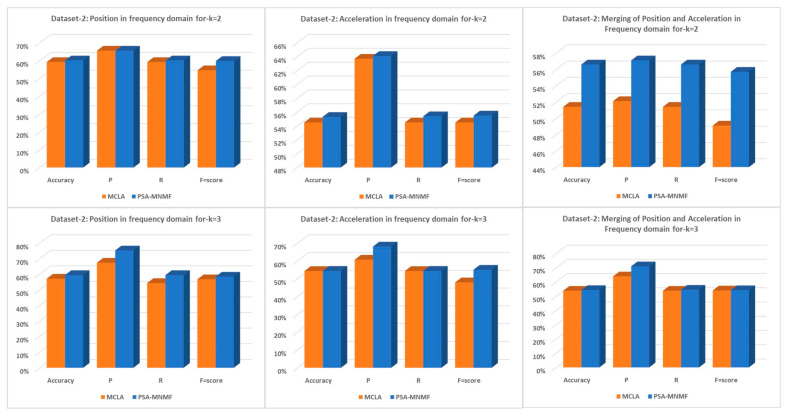
Dataset-2: PSA-MNMF vs. MCLA for k = 2 and k = 3.

For the camera-based dataset-2, all 8 algorithms were implemented and compared for *k* = 2 and *k* = 3 (k is the number of clusters). Each cluster ran 100 times to reduce the variance of the results. Each time, while running, the computational time for *k* = 2 was about 0.33 s for each clustering run and approximately 88.08 s for the consensus clustering run, and *k* = 3, the computational time was about 0.416 s and approximately 73.88 s for the consensus clustering run. Table 11, Table 12 and Table 13 present the results at *k* = 2 on position data, acceleration data, and the merged data on acceleration in frequency and position in the frequency domain for dataset-2, respectively. As shown in Table 11, Table 12, Table 13, Table 14, Table 15 and Table 16, using a two-level and three-level assessment, the proposed PSA-MNMF demonstrated the best performance compared to the baseline clustering methods. We can note from Figure 8 that the proposed PSA-MNMF outperformed the MCLA by achieving higher accuracy, precision, recall, and F-score, thus making it suitable for clinical settings.

## 9. Discussion

This study aimed to determine the severity levels of strokes, evaluate the functions of the affected hands in post-stroke patients, and ultimately automate post-stroke assessment. To analyze a stroke dataset, advanced sensors are required in order to capture stroke movements. One of the strengths of this study was that it deployed an integration of two different sensor technologies—wearable sensor-based and camera-based—that collected the data using the same protocol and selected the same tasks. Both datasets focused on the upper limb in stroke patients and healthy participants while they performed daily living activities. We have proposed a method to estimate stroke survivors’ severity levels—more specifically, to examine the functionality levels of affected hands in stroke patients—by deploying unsupervised learning for the first time to cluster the severity levels in stroke patients. Most studies utilize one to three clusterings; however, to make our results more powerful, 8 clustering methods were implemented for this research. An innovative approach, the PSA-MNMF clustering method, which combines all 8 clustering methods, was proposed. This method was compared with individual clusters as well as with other consensus clustering algorithms such as MCLA. The proposed consensus clustering method offered more robust and consistent results compared to individual clustering and enhanced the performance measurement outcomes compared to other methods. In addition, it must be noted that as per the literature, trunk displacement is one of the compensatory movements considerably presented by stroke patients’ movements. With this knowledge from the literature, we proposed a novel labelling approach where trunk displacement was used to label the severity level in each patient. The frequency domains of position and acceleration and the merged datasets of position and acceleration in the frequency domain were used as part of a unique approach for both datasets in this study. All of the included clustering and consensus clustering models ran 100 times to reduce the variance. The exhaustive search method was applied to find the best combination of clusterings, and the best 10 results among all the possible combinations were selected and reported. The 2-clustering, as well as the 3-clustering, were reported for both datasets. The clustering results using the trunk displacement method were compared with the FMA-UE score that had been reported in the open-source datasets using the standard method, i.e., examination by clinical experts. Then, accuracy, precision, recall, and F-score were reported by comparing the ground truths derived from the FMA-UE score and the clustering labels. The results indicated that for all 12 models (for example, for dataset-1 with 2-clusters, the position in the frequency domain, acceleration in the frequency domain, and the merge data of position and acceleration in the frequency domain, and similarly for 3-clusters and for dataset-2) the PSA-MNMF algorithm presented the highest performance measurements in the form of higher accuracy, precision, recall, and F-score when compared to the individual clustering as well to as the MCLA solver. After the proposed PSA-MNMF method, the MCLA solver, which was the ensemble clustering of all the individuals, demonstrated the highest performance measurement compared to the individual clustering results. The results derived from dataset-1 (which used wearable sensors) showed higher performance when compared to the camera-based systems; this could have been because the camera-based dataset seemed to have more noise compared to the wearable sensor dataset and required more preprocessing. Additionally, the results of the combination of two features, i.e., of position in the frequency domain and acceleration in the frequency domain, did not show any significant changes. The consensus clustering results agreed with the hypothesis that using combinations of several clustering would enhance the final output results. The proposed PSA-MNMF consensus solver presented better results compared to other solvers such as MCLA. Additionally, using the trunk displacement feature as one of the important features to define each cluster showed a promising method. Furthermore, a notable aspect of this study was that it included multiple data collection methods, specifically one that used wearable sensors and one that used camera systems. This choice showcases the versatility and wide-ranging applicability of our proposed method, serves as a key strength, and contributes to the overall richness and depth of this study. In conclusion, from a clinical perspective, the primary contribution of this study is to highlight the importance of transitioning from traditional clinical assessments to employing artificial intelligence (AI)-sensor-based systems for diverse assessments, specifically for those assessments focusing on functional abilities. Furthermore, the study emphasizes the potential of such systems in ultimately evaluating the quality of life among post-stroke patients. This shift in assessment methodologies has the potential to enhance the accuracy, efficiency, and overall understanding of patients’ functional capabilities and well-being, leading to improved care and better outcomes in post-stroke rehabilitation.

## 10. Conclusions and Future Directions

In this paper, motion capture data on stroke patients and healthy subjects were collected from two different clinical universities. The Xsens and Vicon camera datasets were selected for this study since both collected the motion of the upper limb using a shared protocol. However, the inclusion of more datasets with similar protocols and using the same technology while collecting data (such as wearable sensors) could enhance the automated assessment of strokes, as well as any healthcare or rehabilitation planning. Additionally, it must be noted that 4 similar tasks were selected from each motion capture method in this study. However, using the same tasks would enhance the clustering results. The experimental results indicated that consensus clustering enhances cluster output using the novel trunk-displacement labelling method proposed in this study. Future work could investigate limited sensors or markers and compare the results using all upper-limb sensors or markers. Additionally, semi-supervised learning can be examined to automate assessment. Developing an open dataset for stroke patients while they perform FMA-UE tasks could also improve the results when comparing semi-supervised learning with unsupervised learning clustering using FMA scores. For this study, whole actions in participants was considered for finding the mean acceleration; however, looking at smaller steps could be a great future goal for exploration. In addition, for large, collected datasets, gender-based and age-based analysis could be investigated.

## Figures and Tables

**Table 1:** Summary of the studies that used wearable sensors to predict FMA scores.

References	Assessment Tests	Sensors	Result Types	Features	Machine Learning	Purpose
**Meulen et al. (2015)** [26]	Compared with FMA	17 IMUs (Xsens system attached to the body)	Correlation	- Hand position relative to the trunk as well as pelvic region - Quantitative analysis of arm and trunk (Distance)	NA	To assess arm movements and compare them with FMA scores
**Li et al. (2015)** [27]	Compared with Wolf Motor Function Test	2 IMUs attached to the arm and wrist	Correlation	Acceleration and gyroscope	NA	To evaluate motion quality before and after rehabilitation tasks
**Del Din et al. (2011)** [29]	Compared with FMA	Accelerometers to hand, forearm, upper finger, thumb, and sternum.	Prediction	Acceleration	Random Forest	To estimate FMA scores
**Yu et al. (2016)** [21]	Compared with FMA	2 Accelerometers and 7 flex sensors	Prediction	Amp, Mean, RMS, JERK, ApEn ^1^	ELM and SVM to map the result to FMA	To predict FMA scores
**Chaeibakhsh et al. (2016)** [30]	Compared with FMA	5 APDM Opal motion monitoring sensors (APDM Inc., OR, USA). on the sternum, bilateral, dorsal wrists, and bilateral upper arms proximal to the elbow.	Prediction	-Accelerometer and gyroscope sensor, RMSE value, entropy, and dominant frequency	Decision tree and Bootstrap Aggregation Forest	To estimate FMA scores
**Wang et al. (2014)** [31]	Compared with FMA	2 Accelerometer attached to elbow and shoulder	Estimation	Acceleration	a Support Vector Regression	To estimate the FMA scores of shoulder and elbow movements
**Oubre et al. (2020)** [1]	Compared with FMA	9 IMUs (MTw Awinda, Xsens, Netherlands) on the wrist, Sternum	Estimation	Mean velocity Time duration travel distance	DBScan and Regression Model	To estimate FMA scores
**Lee et al. (2018)** [32]	Compared with Functional ability Scale	9 IMUs (MTw Awinda, Xsens, Netherlands) attached to the wrist	Correlation	Velocity	K-means Cluster	- Utilizes kinematic characteristics of voluntary limb movements. -Focuses on the quality of movement in stroke survivors
**Patel et al. (2010)** [28]	Compared with FAS	Accelerometers attached to the hand, forearm, and upper arm	Prediction	Acceleration	Random Forest	To estimate FAS score
**Adans-Dester et al. (2020)** [20]	FMA and FAS	IMU	Upper limb	Displacement, velocity, acceleration, and jerk	Random Forest	Different ADL tasks to evaluate the FAS and FMA scoring

^1^ AMP: amplitude of sensor data; MEAN: mean value of sensor data; RMS: root mean square value of sensor data; JERK: root mean square value of the derivative of sensor data; ApEn: approximate entropy of sensor data.

**Table 2:** A summary of the studies that have used camera-based sensors to predict FMA scores is presented.

References	Assessments Test	Sensors	Results Type	Features	Machine Learning	Purpose
**Kim et al. (2016)** [33]	FMA	Kinect camera data	Correlation	Positions, angles, and the distance between two joints (for instance, hand-shoulder, hand-head and elbow-head)	Artificial Neural Network (ANN)	To develop the FMA tool by utilizing a Kinect camera and to classify the 6 FMA upper extremity tasks
**Mohamed et al.(2021)** [65]	FMA	Kinect V1 and Myo armband	N/A	EMG and position data	SVM	Predicting FMA Scores
**Soe et al. (2019)** [66]	Mallet Clinical Rating Scale	Kinect V1	N/A	Velocity	Rule-based classification	Classifying the Mallet clinical rating scale
**Lee et al. (2017)** [5]	FMA	Kinect, force and resistor sensing hand	Correlation	Acceleration data	Rule-based classifier	Classifying FMA tasks
**Otten et al. (2015)** [34]	FMA	-The Kinect camera pressure sensor (FSR 400 series, Interlink Electronics, Westlake Village, CA, USA) - Glove sensors (a Shimmer inertial measurement unit; IMU, Shimmer, Dublin) - A glove sensor (DG5-VHand glove 3.0, DGTech, Bazzano, Italy)	Prediction	Kinematic features such as finger flexion and extension, joint angle, and supination and pronation of the hand	SVM-Linear SVM-Kernel BNN	To predict 24 out of 33 FMA tasks as 0, 1, or 2 scores.
**Lee et al. (2016)** [35]	FMA	Kinect v2 and force-sensing	Prediction	Joint motion (abduction and adduction, flexion extension, etc.)	Fuzzy-logic classification	To classify movements for FMA prediction purposes. The healthy subjects were used.
**Olesh et al. (2014)** [36]	Arm movements from FMA and Action Research Arm Test	The low-cost motion capture device is called the impulse motion capture system.	Estimation	Joint Angle	Principle Components Analysis (PCA)	Comparing quantitative scores derived from the qualitative clinical scores generated by clinicians

**Table 3:** The activities of daily living tasks selected for this study [82].

Step 1	Step 2	Step 3
Reaching distally and grasping a glass	Drinking for 3 s	Placing it back in the initial position
Reaching distally and grasping a phone	Moving it to the subject’s ear for 3 s	Placing it back in the initial position
Reaching distally and grasping a small cup	Drinking for 3 s	Placing it back in the initial position
Reaching distally and grasping an apple	Pretending to bite	Placing it back in the initial position

**Table 4:** The subjects’ characteristics are described for both wearable sensors and camera-based system.

Characteristics	Mean (SD)/Count (Camera Sensor)	Mean (SD)/Count (Wearable Sensor)
Age	46.77 ± 15.25	61.00 ±10.69
Sex	6 female; 12 male	5 female; 15 male
FMMA-UE	17.75 ± 2.05	46.00 ± 10.16
Affected Hand	12 right; 8 left	11 right; 9 left

**Table 5:** Accuracy, precision, recall, and f-score—Dataset 1 (position in the frequency domain with k = 2).

k = 2	Fuzzy	K-Means	SOM	Gaussian Mixture	DBSCAN	Hierarchical	Spectral	OPTICS	PSA-MNMF
Accuracy	60.4% ± 0.0001	60.7% ± 0.003	60.7% ± 0.036	58.8% ± 0.0001	29.4% ± 0.0001	71.5% ± 0.0002	59.2% ± 0.001	65.5% ± 0.0001	75% ± 0.0001
P	65.8% ± 0.0002	65.9% ± 0.003	66.3% ± 0.024	65.6% ± 0.0001	16.2% ± 0.0001	69.1% ± 0.0002	65.9% ± 0.004	55.1% ± 0.0001	73.9% ± 0.0001
R	60.4% ± 0.0001	60.7% ± 0.003	60.7% ± 0.036	58.8% ± 0.0001	29.4% ± 0.0001	71.5% ± 0.0002	59.2% ± 0.001	65.5% ± 0.0001	75% ± 0.0001
F = score	61.8% ± 0.0001	62.1% ± 0.003	61.9% ± 0.034	60.4% ± 0.0001	14.6% ± 0.0001	68.7% ± 0.0001	60.7% ± 0.001	57% ± 0.0001	74% ± 0.0001

**Table 6:** Accuracy, precision, recall, and f-score—Dataset 1 (acceleration in the frequency domain for k = 2).

k = 2	Fuzzy	K-Means	SOM	Gaussian Mixture	DBSCAN	Hierarchical	Spectral	OPTICS	PSA-MNMF
Accuracy	52.2% ± 0.0001	52.2% ± 0.0002	58.6% ± 0.039	57.1% ± 0.0001	65% ± 0.0001	43.4% ± 0.0001	59.7% ± 0.041	65% ± 0.0001	69.6% ± 0.002
P	70.1% ± 0.0001	70.1% ± 0.0001	68.7% ± 0.008	67.4% ± 0.0001	67% ± 0.0001	69% ± 0.0001	69.1% ± 0.005	48.2% ± 0.0001	70.4% ± 0.001
R	52.2% ± 0.0002	52.2% ± 0.0001	58.6% ± 0.039	57.1% ± 0.0001	65% ± 0.0001	43.4% ± 0.0001	59.7% ± 0.041	65% ± 0.0001	69.6% ± 0.001
F = score	52.2% ± 0.0001	52.1% ± 0.0001	59.7% ± 0.041	58.4% ± 0.0001	67% ± 0.0001	38.4% ± 0.0001	60.9% ± 0.042	54.6% ± 0.0001	68.6% ± 0.001

**Table 7:** Accuracy, precision, recall, and f-score—Dataset 1 (merging of position and acceleration in the frequency domain for k = 2).

k = 2	Fuzzy	K-Means	SOM	Gaussian Mixture	DBSCAN	Hierarchical	Spectral	OPTICS	PSA-MNMF
Accuracy	58.1% ± 0.011	54.4% ± 0.002	61.5% ± 0.033	55.5% ± 0.0001	35.2% ± 0.0001	73.3% ± 0.0001	61.2% ± 0.004	42.5% ± 0.0001	74.3% ± 0.001
P	70.1% ± 0.001	70.1% ± 0.004	70.4% ± 0.011	65.7% ± 0.0001	49.6% ± 0.0001	75.3% ± 0.0001	70.9% ± 0.003	47.8% ± 0.0001	76.4% ± 0.002
R	58% ± 0.011	54.4% ± 0.002	61.5% ± 0.033	55.5% ± 0.0001	35.2% ± 0.0001	73.3% ± 0.0001	61.2% ± 0.004	42.5% ± 0.0001	74.3% ± 0.0019
F = score	59% ± 0.013	54.8% ± 0.003	62.7% ± 0.035	56.9% ± 0.0001	32.2% ± 0.0001	73.7% ± 0.0001	62.5% ± 0.005	44.5% ± 0.0001	74.1% ± 0.001

**Table 8:** Accuracy, precision, recall, and f-score—Dataset 1 (position in the frequency domain with k = 3).

k = 3	Fuzzy	K-Means	SOM	Gaussian Mixture	DBSCAN	Hierarchical	Spectral	OPTICS	PSA-MNMF
Accuracy	48.1% ± 0.016	46.9% ± 0.005	47.8% ± 0.036	44.9% ± 0.0001	13.1% ±0.0001	44.9% ± 0.0001	48.8% ± 0.009	57.5% ± 0.0001	72.1% ± 0.002
P	60% ± 0.016	61.3% ± 0.004	57.5% ± 0.026	61.3% ± 0.0001	16.9% ± 0.0001	63.9% ± 0.0001	56% ± 0.003	48.5% ± 0.0001	79.8% ± 0.001
R	48.1% ± 0.016	46.9% ± 0.005	47.8% ± 0.036	44.9% ± 0.0001	13.1% ± 0.0001	44.9% ± 0.0001	48.8% ± 0.009	57.5% ± 0.0001	72.1% ± 0.002
F = score	51.1% ± 0.012	50% ± 0.005	50.3% ± 0.034	47.1% ± 0.0001	14.1% ± 0.0001	46.4% ± 0.0001	51.3% ± 0.008	44.3% ± 0.0001	72.3% ± 0.001

**Table 9:** Accuracy, precision, recall, and f-score—Dataset 1 (acceleration in the frequency domain for k = 3).

k = 3	Fuzzy	K-Means	SOM	Gaussian Mixture	DBSCAN	Hierarchical	Spectral	OPTICS	PSA-MNMF
Accuracy	46.2% ± 0.0001	46.5% ± 0.007	45% ± 0.026	48% ± 0.0001	22.1% ± 0.0001	43.6% ± 0.0001	38% ± 0.0002	51.5% ± 0.0001	67.30% ± 0.007
P	67.4% ± 0.0001	67.7% ± 0.004	56.6% ± 0.072	50% ± 0.0001	65.8% ± 0.0001	69.5% ± 0.0001	68.6% ± 0.0002	38.1% ± 0.0001	70.30% ± 0.03
R	46.2% ± 0.0001	46.5% ± 0.007	45% ± 0.026	48% ± 0.0001	22.1% ± 0.0001	43.6% ± 0.0001	38% ± 0.0002	51.5% ± 0.0001	67.3% ± 0.007
F = score	47.3% ± 0.0001	47.5% ± 0.008	45.7% ± 0.03	48.6% ± 0.0001	12.8% ± 0.0001	43% ± 0.0001	34.3% ± 0.0002	41.9% ± 0.0001	66% ± 0.007

**Table 10:** Accuracy, precision, recall, and f-score—Dataset 1 (merging position and acceleration in the frequency domain for k = 3).

k = 3	Fuzzy	K-Means	SOM	Gaussian Mixture	DBSCAN	Hierarchical	Spectral	OPTICS	PSA-MNMF
Accuracy	46.2% ± 0.0001	46.2% ± 0.005	46.6% ± 0.03	48.7% ± 0.003	22.6% ± 0.0001	46.9% ± 0.0001	40.3% ± 0.0001	46.5% ± 0.0001	67.9% ± 0.04
P	67.7% ± 0.0001	68.8% ± 0.007	57.5% ± 0.062	53.6% ± 0.006	27.2% ± 0.0001	70.3% ± 0.0002	67.5% ± 0.0002	35% ± 0.0001	70.80% ± 0.053
R	46.2% ± 0.0001	46.2% ± 0.005	46.6% ± 0.03	48.7% ± 0.003	22.6% ± 0.0001	46.9% ± 0.0002	40.3% ± 0.0001	46.5% ± 0.0001	67.9% ± 0.01
F = score	46.4% ± 0.0001	46% ± 0.007	47.3% ± 0.033	49.8% ± 0.003	14.8%± 0.0001	45.4% ± 0.0001	37.6% ± 0.0001	39.2% ± 0.0001	66.20% ± 0.03

**Table 11:** Accuracy, precision, recall, and f-score—Dataset 2 (position in the frequency domain for k = 2).

k = 2	Fuzzy	K-Means	SOM	Gaussian Mixture	DBSCAN	Hierarchical	Spectral	OPTICS	PSA-MNMF
Accuracy	50.6% ± 0.021	49.1% ± 0.001	47.6% ± 0.065	56.9% ± 0.001	35.9% ± 0.0001	40.8% ± 0.0001	48.5% ± 0.0001	45.2% ± 0.0002	60.1%± 0.001
P	50.7% ± 0.022	49.1% ± 0.001	47.6% ± 0.067	57% ± 0.001	35.7% ± 0.0001	40.8% ± 0.0002	48.5% ± 0.0001	25.9% ± 0.0010	65.6%± 0.003
R	50.6% ± 0.021	49.1% ± 0.001	47.6% ± 0.065	56.9% ± 0.001	35.9% ± 0.0001	40.8% ± 0.0001	48.5% ± 0.0002	45.2% ± 0.0001	60.1%± 0.001
F = score	50.1% ± 0.017	48.9% ± 0.001	47.3% ± 0.065	56.7% ± 0.001	35.7% ± 0.0001	40.7% ± 0.0001	48.4% ± 0.0002	31.4% ± 0.0002	59.8%± 0.001

**Table 12:** Accuracy, precision, recall and f-score—Dataset 2 (acceleration in the frequency domain for k = 2).

k = 2	Fuzzy	K-Means	SOM	Gaussian Mixture	DBSCAN	Hierarchical	Spectral	OPTICS	PSA-MNMF
Accuracy	39% ± 0.0001	38.9% ± 0.003	38.4% ± 0.035	36.2% ± 0.0001	46.2% ± 0.0001	30.5% ± 0.0001	49.4% ± 0.0001	48.1% ± 0.0001	55.3% ± 0.0008
P	38.4% ± 0.0001	38.4% ± 0.003	38.1% ± 0.034	36.2% ± 0.0002	45% ± 0.0001	26.6% ± 0.0002	24.8% ± 0.0001	24.4% ± 0.0002	64.1% ± 0.0007
R	39% ± 0.0001	38.9% ± 0.003	38.4% ± 0.035	36.2% ± 0.0001	46.2% ± 0.0001	30.5% ± 0.0001	49.4% ± 0.0001	48.1% ± 0.0001	55.4% ± 0.0008
F = score	38.2% ± 0.0001	38.2% ± 0.003	38% ± 0.033	36.2% ± 0.0001	43.1% ± 0.0001	27.5% ± 0.0001	33% ± 0.0001	32.4% ± 0.0001	55.5% ± 0.0008

**Table 13:** Accuracy, precision, recall, and f-score—Dataset 2 (merging position and acceleration in the frequency domain for k = 2).

k = 2	Fuzzy	K-Means	SOM	Gaussian Mixture	DBSCAN	Hierarchical	Spectral	OPTICS	PSA-MNMF
Accuracy	38.7% ± 0.0001	39% ± 0.002	37.9% ± 0.046	37.5% ± 0.0001	47.4% ± 0.0001	31.5% ± 0.0002	49.4% ± 0.0001	56.1% ± 0.0001	56.6% ± 0.009
P	38.2% ± 0.0001	38.5% ± 0.002	37.6% ± 0.046	37.5% ± 0.0002	47.1% ± 0.0002	28.4% ± 0.0001	24.8% ± 0.0002	51.9% ± 0.0001	57.1% ± 0.0019
R	38.7% ± 0.0002	39% ± 0.002	37.9% ± 0.046	37.5% ± 0.0001	47.4% ± 0.0002	31.5% ± 0.0001	49.4% ± 0.0001	56.1% ± 0.0001	56.6% ± 0.001
F = score	38% ± 0.0001	38.3% ± 0.002	37.5% ± 0.046	37.5% ± 0.0001	46.3% ± 0.0002	29% ± 0.0002	33% ± 0.0002	47.4% ± 0.0001	55.7% ± 0.002

**Table 14:** Accuracy, precision, recall, and f-score—Dataset 2 (position in the frequency domain for k = 3).

k = 3	Fuzzy	K-Means	SOM	Gaussian Mixture	DBSCAN	Hierarchical	Spectral	OPTICS	PSA-MNMF
Accuracy	50.6% ± 0.019	49.1% ± 0.001	47.7% ± 0.051	56.8% ± 0.002	35.9% ± 0.0	40.8% ± 0.0	48.5% ± 0.013	45.2% ± 0.0	59.41% ± 0.004
P	50.6% ± 0.02	49.1% ± 0.001	47.7% ± 0.052	56.9% ± 0.002	35.7% ± 0.0	40.8% ± 0.0	48.5% ± 0.021	25.9% ± 0.0	75.3% ± 0.0001
R	50.6% ± 0.019	49.1% ± 0.001	47.7% ± 0.051	56.8% ± 0.002	35.9% ± 0.0	40.8% ± 0.0	48.5% ± 0.013	45.2% ± 0.0	59.41% ± 0.004
F-score	50.1% ± 0.016	49% ± 0.001	47.5% ± 0.05	56.6% ± 0.001	35.7% ± 0.0	40.7% ± 0.0	48.4% ± 0.016	31.4% ± 0.0	58.4% ± 0.0001

**Table 15:** Accuracy, precision, recall, and f-score—Dataset 2 (acceleration in the frequency domain for k = 3).

K = 3	Fuzzy	K-Means	SOM	Gaussian Mixture	DBSCAN	Hierarchical	Spectral	OPTICS	PSA-MNMF
Accuracy	27% ± 0.009	26.4% ± 0.01	29.1% ± 0.043	26.6% ± 0.022	23.8% ± 0.0001	30.3% ± 0.0001	48.9% ± 0.0001	14% ± 0.0001	54.4% ± 0.0001
P	51% ± 0.007	51.1% ± 0.004	47.4% ± 0.06	50.8% ± 0.009	24.3% ± 0.0001	58.5% ± 0.0001	24.8% ± 0.0001	39.9% ± 0.0001	68.1% ± 0.0001
R	27% ± 0.009	26.4% ± 0.01	29.1% ± 0.043	26.6% ± 0.022	23.8% ± 0.0001	30.3% ± 0.0001	48.9% ± 0.0001	14% ± 0.0001	54.4% ± 0.0002
F-score	33.9% ± 0.008	33.2% ± 0.007	35.8% ± 0.047	33.7% ± 0.018	24.1% ± 0.0001	32.3% ± 0.0001	32.9% ± 0.0001	17% ± 0.0001	55% ± 0.0002

**Table 16:** Accuracy, precision, recall, and f-score—Dataset 2 (merging of position and acceleration in the frequency domain for k = 3).

k = 3	Fuzzy	K-Means	SOM	Gaussian Mixture	DBSCAN	Hierarchical	Spectral	OPTICS	PSA-MNMF
Accuracy	27.9% ± 0.017	26% ± 0.016	28.7% ± 0.04	33.1% ± 0.002	32% ± 0.0001	30% ± 0.0001	48.6% ± 0.0001	43.4% ± 0.0001	54.2% ± 0.009
P	49.4% ± 0.015	50.6% ± 0.009	47.7% ± 0.052	46.8% ± 0.004	25.6% ± 0.0001	58.7% ± 0.0001	24.6% ± 0.0001	33.3% ± 0.0001	70.9% ± 0.015
R	27.9% ± 0.017	26% ± 0.016	28.7% ± 0.04	33.1% ± 0.002	32% ± 0.0001	30% ± 0.0001	48.6% ± 0.0001	43.4% ± 0.0001	54.4% ± 0.07
F = score	34.4% ± 0.014	32.8% ± 0.013	35.7% ± 0.043	38.4% ± 0.003	28.5% ± 0.0001	32.4% ± 0.0001	32.6% ± 0.0001	31.5% ± 0.0001	54.1% ± 0.002

## Data Availability

The camera-based data open-source are available in section MDPI Research Data Policies at https://dataverse.harvard.edu/file.xhtml?persistentId=doi:10.7910/DVN/FU3QZ9/PILNU7&version=4.0, and the Wearable sensor open dataset (Xsens dataset) are available at https://zenodo.org/record/3713449#.Y9RC0nbMI2x. (accessed on 12 March 2021).
